# Art therapy practices in museum education: A mini review

**DOI:** 10.3389/fpsyg.2022.1075427

**Published:** 2023-01-20

**Authors:** Zhizi Wei, Chaofang Zhong, Yunteng Gao

**Affiliations:** ^1^College of Teachers Education, Zhejiang Normal University, Jinhua, Zhejiang, China; ^2^Shandong First Medical University, Jinan, Shandong, China

**Keywords:** art therapy practices, integrated art therapy, museum education, psychological anxiety, virtual reality

## Abstract

This article reviews the potential functions and approaches of museum education in alleviating psychological anxiety, particularly the psychological anxiety experienced by adolescents during the COVID-19 pandemic. We outline the main forms of museum education, highlighting how it supports the potential functions of art therapy for psychological anxiety. Thereafter, we review the representative research on museum art therapy practice for different populations to invite discussion, dialogue, and awareness of future directions for museum education and suggest gaps in the research that require further study.

## 1. Introduction

Museums are important informal learning environments. The traditional one-way educational role of museums is changing, driven by new museology and related participatory practice research (Dewey, 1916; Vergo, 1989; Sandell, 1998; Davallon, 1999; Marstine, 2006; Mairesse and Desvallées, 2007; Simon, 2010; McSweeney and Kavanagh, 2016). Museums are also a key asset for community well-being, contributing to local health and welfare goals and maintaining public well-being (Lackoi et al., 2016). Indeed, a paradigm shift has occurred from simply displaying rare art collections to enriching visitor engagement; museums now offer art prescription programs and “hard-to-reach” museum audiences are engaged through museum education. These programs are designed to allow participants to communicate non-verbally through art, craft, photography, or design. In curating or participating in museum exhibitions to creatively express themselves, participants’ selves are nurtured (Morse and Munro, 2018). Consequently, museums are interacting and influencing people in a richer way and asserting their role as a public service, with education at its center. Museum education can be defined as a set of values, concepts, knowledge, and practices designed to ensure visitors’ development: It is a cultural adaptation process that relies on pedagogy, development, practice, and acquiring new knowledge (Desvallées and Mairesse, 2010, p: 31).

The COVID-19 pandemic continues to pose a global challenge to mental health. The World Health Organization (2019) warns that a lack of mental health care is a major cause of the global disease burden. One-fifth of children and adolescents worldwide have mental health problems. Their unmet psychosocial needs are exacerbated by war, adversity, discrimination, disease, and natural disasters. Surveys show an increasing trend in psychological distress among adolescents, manifesting as learning and somatic anxiety, and easily becoming sensitive and fearful (Qin et al., 2021). Such problems can seriously interfere with daily functioning (Bhosale et al., 2015; Veldman et al., 2015) and increase the risk of underperforming in school (Veldman et al., 2015). Hence, there is a greater need for psychotherapy to reduce the incidence of psychological problems (Li et al., 2020).

This mini review aims to invite discussion, dialogue, and recognition of the potential functions of museum education in therapy to expand the range of museum education services, explore the possibilities of museum involvement in art therapy through representative case studies of this practice with diverse populations, and highlight the effectiveness of virtual reality (VR) museum education in providing distance therapy for adolescents. Our work in museums inspired us to better understand the literature on the use of art therapy approaches in museums.

## 2. Materials and methods

We screened representative literature for different populations in the Web of Science Core Collection database. Web of Science is a comprehensive multidisciplinary core journal database. Web of science database has the world’s largest and most comprehensive academic information resources, covering more than 12,000 academic journals in natural science, engineering, biomedicine, social science, art, humanities and other disciplines. This article uses the Web of Science Core Collection [Science Citation Index Expanded (SCIE) and Social Sciences Citation Index (SSCI)] database as the data source. The search formula is: [TS = ('art *' OR 'artistic *') and ('treatment *' OR 'cure *' OR 'treat *' OR 'therapy *')] and ('museum *' OR 'museums *') and ('education *' OR 'educational *'). We did not restrict the publication year; however, the source language was limited to English. The search yielded 10 representative peer-reviewed articles citing museum education and art therapy practices, including theoretical and critical perspectives (Treadon et al., 2006; Salom, 2011; Ioannides et al., 2021), ethnography, qualitative analysis, and clinical treatment cases (Colbert et al., 2013; Mangione, 2013; Bennington et al., 2016; Zarrabi et al., 2020; Armstrong et al., 2021; Price et al., 2022; Wallen and Docherty-Hughes, 2022). Specific practices for different groups are reported, covering different benefits for anxiety, traumatic memories, self-disappointment, geriatric groups, Alzheimer’s disease, chronic illness, and psychiatric patients (Table 1).

**Table 1:** Key findings.

Article Title	Author (year)	Treatment Topics	Research content	Key findings
Caring Spaces: Individual and Social Wellbeing in Museum Community Engagement Experiences	Wallen and Docherty-Hughes (2022)	Self-reflection	It is only possible to interpret art in a safe way through personal experience when their peers understand the challenges of living with mental illness or the reality of becoming homeless with safety knowledge.	The positive impact on the overall well-being of the “caring spaces” generated through museum community engagement work is achieved through a process of deep critical reflection, which enhances self-esteem and self-confidence, and raises participants’ awareness of positioning ontology in the context of broader social inequalities and identity issues. Museum community engagement programs, when practiced and experienced as “spaces of care,” play a key role in enhancing participants’ own personal and social well-being, particularly in identifying long-term educational and self-worth legacies.
Exploring Trauma Responsive Educational Practices in a Museum	Price et al. (2022)	Trauma Responsive Education Practice (TRE)	How one museum learned about the presence and impact of trauma by exploring the framework developed by the Trauma Responsive Educational Practices (TRE) program.	They needed more support to adapt to the unique environment of the museum; evoked memories of personal trauma for some staff members, so they needed to rethink implementation.
Weaving Trauma Awareness into Museum Education	Armstrong et al. (2021)	Art Therapy for Trauma	This paper explores the dynamics of trauma-informed approaches to interacting with art, specifically detailing methods that can create new cognitive, emotional, and sensory experiences while outlining the key principles of Trauma-Aware Art Museum Education (T-AAME) as it relates to visitors.	Trauma-aware museum educators have a therapeutic role in helping visitors reintegrate into their lives. The dynamics of a trauma-aware approach to interacting with art are explored.
The “Third Object” in Palliative Care Education: Impact of a Novel Art Museum–Based Curriculum to Foster Reflection, Self-Awareness, and Teamwork Among a Multidisciplinary Palliative Care Team (S748)	Zarrabi et al. (2020)	Art Theme Experience	MBE pedagogy constructed around the themes of pain, healing, dignity, complexity, and legacy MBE pedagogy constructed around the themes of pain, healing, dignity, complexity, and legacy	Four themes were identified that exemplify the value of this educational experience: (1) using art as a “proxy” in a neutral setting to safely access emotions and work through difficult situations, (2) appreciating the value of experiential immersion in clinical development, (3) discovering the power of the multidisciplinary palliative care team, and (4) shaping work-life balance.
Contemporary artworks as transformational objects in art psychotherapy museum group work	Ioannides et al. (2021)	Art Psychotherapy	It examined two group art psychotherapy projects held at the National Museum of Contemporary Art (EMST) in Athens in 2017. It focused on three contemporary artworks from the EMST collection, by Kimsooja, Ilya Kabakov, and Sophia Kosmaoglou, and how working with these groups could be explored through object relations theory.	By linking contemporary art to existing object relations theory, contemporary art benefits when practicing psychotherapy in a museum setting. We conclude that professionals who run the museum and gallery-based psychotherapy groups will find the contributions of object relations theory and contemporary art beneficial in this way. Art psychotherapy can be beneficially implemented in a museum setting.
Art therapy in art museums: Promoting social connectedness and psychological well-being of older adults	Bennington et al. (2016)	Art museum education increases well-being and social connection work	Explores the phenomenological perspectives of older adults who participate in museum art therapy groups and attempts to understand how the functions of art proposed by de Botton and Armstrong (2013) are reflected in the art and writing of older adults who visit museums. Museums with art therapists. Emergent categories in art and writing are integrated into de Botton and Armstrong’s broader themes of art functioning. Outcomes related to mental health, quality of life, and perceived social support are also addressed.	We examined the use of art museums as a therapeutic tool for older adults. - Art museums offer a safe exploration of emotions, thoughts, and memories. - Qualitative data show increased well-being and social connectedness. - This study supports previous research on the therapeutic value of art museums.
Access to what? Alzheimer’s disease and esthetic sense-making in the contemporary art museum	Mangione (2013)	Dialogue activities for art museum education	Using ethnographic fieldwork and in-depth interviews at the two Metropolitan Museums of Art outlets, we examined how educators and participants constructed the benefits of art museum programs for Alzheimer’s disease patients.	Participating in recreational activities such as museum tours is an important way to stay on track in the face of chronic illness. Educators use relativist language to structure the arts to facilitate interaction, providing opportunities for greater dialogue between culture and the sociological study of health.
Opening the doors of art museums for therapeutic processes	Treadon et al. (2006)	Evaluation of art museums as effective therapeutic tools	An overview of the establishment of art museums and the history of art museum education. A similar examination of previous art therapy programs, and a framework for using art museums as effective therapeutic tools. The study concludes with a description and evaluation of a pilot project to incorporate art museums and their artifacts into art therapy.	The Art Museum is a new venue for art therapy. Museums can be valuable to art therapists by providing a wealth of resources for clients and art therapy. Art museum educators have a role in helping art therapists understand how to approach art museums for use by their clients.
The art-gallery as a resource for recovery for people who have experienced psychosis	Colbert et al. (2013)	Psychiatric Rehabilitation Intervention	To understand whether an art gallery-based group helped to modify key psychiatric narratives in participants’ personal narratives, promoting recovery, well-being, and a subjective sense of social inclusion.	The findings suggest that some individuals used art-related concepts to modify dominant narratives in their personal narratives. Community narratives emerged from the groups about different employee-client relationships characterized by recognition, commonality, friendship, and sincerity. The intervention was described as promoting recovery and well-being primarily through achievement and was described as more successful in addressing the bonding of social capital than bridging it. Art gallery-based interventions show some promise of providing a safe haven where people with mental illness can engage in a recovery-oriented approach to mental health care, where a different kind of staff-client relationship may emerge.
Reinventing the setting: Art therapy in museums	Salom (2011)	Four metaphorical roles that museums can play to promote therapeutic goals	Using two cases that explore whether the life stages, museums, their environments, and the objects they care about can become effective allies in art therapy.	This paper proposes four metaphorical roles for museums to promote therapeutic goals. These roles are: museum as co-leader, as a group, as self, and as an environment. Examples of their practical implementation in therapy are presented.

## 3. Results

### 3.1. Activity approach and therapeutic function of museum education

The educational purpose of a museum influences all the activities that take place within it and determines its therapeutic function. The visit experience can lead to long-term introspective and cognitive outcomes, especially in social awareness (DeWitt and Storksdieck, 2008; Annechini et al., 2020).

**Figure 1:**
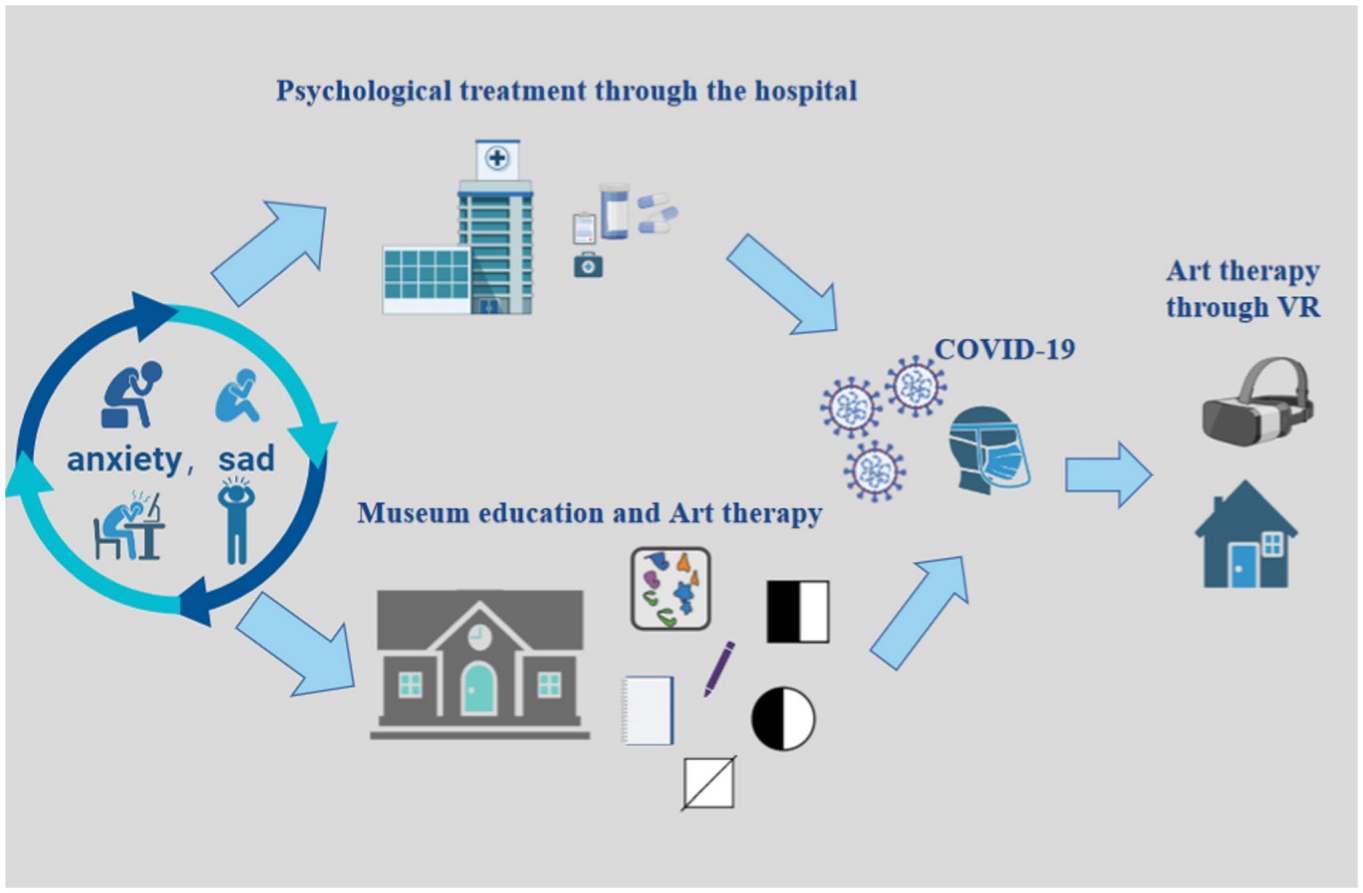
Generalized diagram of museum education and art therapy.

During the pandemic, various populations developed psychological problems such as anxiety, depression, and insomnia due to stress. Some patients experienced suicidal ideation or acute psychiatric disorders. Compared with traditional therapeutic interventions, the museum serves as a supportive place that embraces social inclusion, promotes cohesion by being open to people from all walks of life, and provides a space for reflection on ideas that combat discrimination and enhance well-being. These responsibilities demonstrate the value of museums as healing places (Ioannides, 2016; Figure 1). As Annechini et al. (2020) argue, museums have restorative effects on children’s psychology.

### 3.2. Potential functions of museum education

Museum practices, museum professionals, and academics have gradually expanded the concept of education in museums for learning activities in museum settings (Allard and Boucher, 1998; Hein, 2002; Leinhardt et al., 2003). Current museum education practices can be categorized into universal, heuristic, exploratory, living, physical experience, and artificial intelligence educational activities (Table 2).

**Table 2:** Main forms of museum educational activities.

**Education form definition**	**Activities conducted/content of activities**	**Remarks**
Universal, lifelong educational activities	All people share museum resources and plan different types of educational activities for different categories of visitors. To expand the educational function, we take the initiative to serve in schools and community activities.	Traveling exhibitions, teaching aids and materials lending services, etc.
Inspiring and entertaining educational activities	Instead of the traditional window display and solemn atmosphere, museum displays and activities are now more active and diversified, replacing the passive learning method of only “seeing” with many models, audio-visual aids, games, and various participatory and interactive designs in open displays.	Scavenger hunt competition, role play, audio-visual appreciation, demonstration performance, etc.
Self-directed, exploratory educational activities	Unlike traditional school-based learning, today’s museums offer guided tours, tour information systems, and activity sheets, as well as themed activities, to encourage audiences to find answers and gain a sense of accomplishment and self-confidence through personal experience.	“Discovery Room,” “Self-Awareness Room,” or other theme-based exploration activities.
Lifestyle educational activities	Activities are designed to deepen the audience’s impressions and enhance their learning, not only by exploring past events but also by focusing on the way participants’ perceptions and experiences are formed.	Guidance in learning to live, face prejudice, and violence, etc.
On-site, physical experience-based educational activities	Through the physical scenery, scenario shaping, or site restoration in three dimensions, human history or art from a distant time and space can be recreated, leaving the audience with an immersive and moving experience.	Cultural scene restoration activities in village museums.
Artificial intelligence-style educational activities	The use of artificial intelligence in museum education is a new trend in the development of the interconnected era. Artworks can reproduce multiple spatial and temporal dimensions through artificial intelligence, which not only brings more direct sensory enjoyment, but also provides audiences with an immersive art experience. Although this form is in the early stages of development, it will have a broader impetus for the development of museum education forms.	Common pop-up screen presentation of multiple virtual space dimensions and intelligent art element data analysis and evaluation.

### 3.3. The art therapy function of museum education

Museums have noted the usefulness of art therapy for improving mental health (Stickley and Eades, 2013) and can work with the health care or higher education sectors. Multisectoral collaboration is effective in improving participants’ mental health and well-being (Docherty-Hughes et al., 2020). However, the potential for art therapy to expand into new areas has not yet been fully recognized and its spiritual and mental health improvement effects not fully explored. There is an urgent need to strengthen the support given to museums and related cultural institutions for art therapy work, which can be widely applied to significantly help patients embarrassed about their disease or resisting treatment.

The combination of museum education and art therapy plays a vital role in improving psychological anxiety, and art therapy enables individuals to make connections between internal and external reality (Treadon et al., 2006; Shaer et al., 2008; Colbert et al., 2013; Betts et al., 2015; Salom, 2015; Ioannides, 2016; Coles and Harrison, 2018; Coles et al., 2019; Coles, 2020). This combination meets the needs of contemporary society and demonstrates the potential benefits of museums as non-traditional tools for art psychotherapy, providing help through meaningful connections and allowing individuals to “reflect on and share their past” (Salom, 2011, p: 83).

Museum educators (Froggett et al., 2011; Colbert et al., 2013) and art therapists (Treadon et al., 2006) believe that art therapy in museums has become an increasingly important endeavor, especially for mental health support (Treadon et al., 2006). The most popular types of museum programs are those designed to allow participants to express themselves creatively through art, craft, photography, design, curation, or participation in museum exhibitions (Morse, 2021). Museums help participants reflect on lived experiences, reconcile the past, and reconstruct narratives *via* educational and creative arts programs (Camic and Chatterjee, 2013). They provide a potential therapeutic opportunity (Salom, 2008) and a “caring” space (Morse, 2021) and offer opportunities for self-development, spiritual and artistic growth, and social connection (Roberson, 2011).

Additionally, art “can help guide, exhort, and comfort audiences to become better versions of themselves” (de Botton and Armstrong, 2013). The potential benefits of art therapy include memory, hope, grief processing, rebalancing, self-understanding, growth, and appreciation. Thus, art therapy in a museum setting may help adolescents increase their sense of belonging and support self-reflection and other psychological needs. Activation of autobiographical memories is associated with feelings of well-being (O’Rourke et al., 2011), and this function of art can be combined with the therapeutic effects of reminiscence. The process of sharing memories and praising one another supports group cohesion and improves mental health (Wadensten, 2005; Chiang et al., 2010). Thus, museum-based art therapy can potentially alleviate psychological anxiety among adolescents.

#### 3.3.1. Mental restoration

Museum-based art therapy facilitates mental restoration by relieving physical tension and mental anxiety. Many people visit museums for relaxation and physical rejuvenation because museums can repair “attention fatigue” and provide “restorative environment” conditions (Packer, 2008). They provide a place where young people can explore, unrushed and away from their usual environment and daily routines.

#### 3.3.2. Self-reflection

Self-reflection is an important component of mental health, including the process of identifying, thinking about, and making sense of one’s feelings, experiences, and ideas. “Adolescents naturally engage in the construction of individual meaning and self-exploration at museums by reflecting on the situations they face and engaging in discussions with others” (Wang, 2018, p:5).

#### 3.3.3. Physical and mental balance

Museum-specific artwork can help people shift their focus from their anxieties to their bodies; they become aware of their existence, perceive the value of their being, and return to their bodies and minds. From the essence of “things,” museums reflect both the uniqueness of the times and the differences in history. From the existence of “human beings,” museums show that greatness and fragility have coexisted for generations of human beings, creating an atmosphere wherein young people can express different ideas and thoughts, and providing a “laboratory” to understand the close connection between “things” and “people.”

### 3.4. Approaches of art therapy practice in museums

Art can provide “a visual link to a personal exploration of past and present experiences” (Stephenson, 2006, p: 24); this is particularly important because the activation of autobiographical memory is associated with a sense of well-being (O’Rourke et al., 2011). As viewing art provides a unique psychological experience, art therapy in museums offers the opportunity to restore health and well-being, especially for patients suffering from mental health issues (Colbert et al., 2013). De Botton and Armstrong (2013) describe the function of memory in art as a way of holding on to things deemed precious and fleeting.

The three theoretical perspectives highlight the role museums play in the art therapy process and the different supports they provide to promote therapeutic goals (Treadon et al., 2006; Salom, 2011; Ioannides et al., 2021). Ioannides et al. (2021) notes that contemporary artwork can serve as therapeutic transformative objects that can evoke a stronger sense of self-identity and help restore mental health. Salom (2011) summarizes four metaphorical roles museums play in therapy: co-leader, group, self, and environment.

Treadon et al. (2006) refers to the richness of artwork and cultural resources that art therapists provide to participants through museum education, emphasizing museums’ efforts to engage non-traditional populations and the facilitative role of educators in art therapy. Similarly, Ioannides et al. (2021) emphasizes the intrinsic quality of specific artwork as an evocative element for individual participants. She outlines how participants can express their feelings, discuss difficult issues, and internalize the cultural world through contemporary artwork (works displayed in the museum as well as artwork created by participants) to understand, explore, and reveal their different aspects. This approach allows participants’ emotional experiences to be better understood, facilitating their relationship with the external world and stimulating the presence of self-states in their spiritual lives because they contain “a layer of meaningful connections that stimulate” associations. These associations enable individuals experiencing personal or mental health problems to “recall and share their past” (Salom, 2011, p: 83). Salom (2011) suggests that curators pave the way for personal expression and transformation through interactive art exhibitions (Treadon et al., 2006). Museums are interested in increasing outreach by integrating clinical and educational knowledge (Linesch, 2004).

There are also studies that focus on people with Alzheimer’s disease, chronic disease or mental illness. Wallen and Docherty-Hughes (2022) emphasize the critical role of museum education in enhancing the self-esteem and social well-being of individuals with psychological problems through “caring spaces.” They established the Self-Reflection Project’s community-based museum art therapy program, highlighting the role of museum art therapy in lifelong learning and leveraging heritage values, including overall well-being and individual identity. During the pandemic, one museum learned about the impact of trauma by exploring the framework developed by the Trauma Responsive Educational Practices (TRE) program. The authors conclude that additional support is needed to adapt the program to the museum’s unique environment. At the Rubin Museum of Art, workers began to care about the same issues, and in their Nepalese art collection, there are some content that is very suitable for self-reflection. Now, the museum is planning to restart their meditation podcasts and arrange some of the learning sessions to people affected by the new corona, which will include some meditative works of art, such as a gilded statue of the 13th-century Indian goddess Durga, and a 16th-century Buddha painting in which the Buddha is meditating while the demonic army is attacking from below. The Queens Museum in New York, the United States, offers an online art therapy program every Thursday, allowing community participants to share their creations through Zoom and use paintings and poetry to discuss life before and after the pandemic. The Metropolitan Museum of Art of the United States is also launching a free art therapy program. The museum has designed the exhibition space as a safe space with trauma awareness, using the same practices used in the aftermath of the 9 / 11 terrorist attack that year. The artwork on display tries to alleviate the anxiety or sadness of visitors in the face of the epidemic. Walter Enriquez, a 75-year-old New York resident, has lost many friends and neighbors due to the epidemic. He spends 30 min sitting in front of the computer every Thursday, drawing on paper with colored pencils and pens. Enriquez said: ‘Before taking part in the programme I felt very lonely, but now, I can study artistic creation. We can ‘t enjoy life as before, but art can help us capture the past, regain positive experiences, and get through pain and sadness. ‘Bouvayer also said that in the past, doctors rarely did not have to worry about any side effects when prescribing, and now there is such a development is very touching. In her opinion, the doctor ‘s establishment of ‘museum prescription notes ‘is a way to connect with patients on an emotional level. ‘We always ask ourselves: What else can I do? From now on, we can at least provide ‘happy moments ‘, ‘.

In addition, the role of different art therapy methods in museum education was also investigated. Armstrong et al. (2021) investigated the dynamics of trauma-informed approaches to interacting with art, specifically approaches that create new cognitive, emotional, and sensory experiences. Outlining key visitor-related trauma-informed art museum education (T-AAME) principles, they report that museum educators can play a therapeutic role in helping visitors reintegrate into their lives. The mind, brain, and education (MBE) pedagogy, constructed by Zarrabi et al. (2020) around the themes of pain, healing, dignity, complexity, and legacy, identifies four themes that reflect the value of the educational experience: (1) using art in a neutral setting as a “proxy” to safely access emotions and work through difficult situations, (2) appreciating the value of experiential immersion in clinical development, (3) discovering the power of the multidisciplinary palliative care team, and (4) shaping work-life balance. Colbert et al. (2013) assessed participants’ mental health and explored recovery-oriented approaches to mental health care through an art gallery intervention, finding that art galleries can provide a safe haven that promotes recovery, well-being, and a sense of social inclusion.

Bennington et al. (2016) examined the use of art museums as a therapeutic tool for older adults from a phenomenological perspective, demonstrating that they offer curricular explorations of emotions, thoughts, and memories, increasing well-being and social connectedness. Another study on how institutional meanings emerge at the local level, revealed how educators and participants constructed the benefits of art museum programs for people with Alzheimer’s disease, providing an opportunity for more dialogue between the sociological studies of culture and health (Mangione, 2013).

In summary, the review highlights the therapeutic potential of museum education and art therapy to improve mental health and promote recovery, well-being, and a sense of social inclusion among different populations. However, no existing research has focused on how museum-based art therapy can ameliorate psychological anxiety issues in adolescents or the challenges of working with clinical diagnostic categories of adolescents in art therapy research and clinical practice from a museum education perspective.

## 4. Discussion

With the increasing attention paid to Art Therapy in recent years, the Montreal Art Gallery, Canada ‘s oldest art museum, has received a large donation funding. In 2016, the museum ‘s education and art therapy functions were expanded; in 2017, it became the first museum in North America to employ full-time art therapists. In 2018, it became the first art museum to accept local doctors to open ‘museum prescription notes ‘, allowing patients to visit for free and even book art therapists in the museum. In the United States, some museums preparing to reopen are seeing art healing as their new direction. The Queens Museum of Art offers a weekly online art therapy program that encourages people to pick up a paintbrush to express their lives and feelings. The Metropolitan Museum of Art in New York has prepared a list of works of art to help spectators ease anxiety about the new corona after the museum reopens. The Cincinnati Museum of Art in Ohio trained a group of volunteers to teach art healing techniques. These measures were inspired by the Montreal Museum of Art in Canada, which is the first art institution in North America to hire an art therapist.

By reviewing peer-reviewed literature, this paper discusses how museum practitioners can develop a program of activities to promote the potential therapeutic function of museums and integrate art therapy approaches into practice. The literature suggests that museum therapy practices are strongly associated with participants’ sense of self-identity, well-being, and social well-being, all of which can help alleviate anxiety. This review provides a preliminary understanding of the issues involved in museum education practices that require further examination, including the propensity of different museum learning programs to improve psychological problems among different groups.

### 4.1. New directions for museum education

VR is widely used in museums to augment or simulate artwork and artifacts to create more vivid and immersive learning environments. In the last decade, the boom in digital technology has driven many museums to publish collections online, and much related research has been undertaken by ARCO, SCULPTEUR in Europe, VMC in Canada, Sci Center and Bio Learn in the US (Corbit and De Varco, 2000), and similar programs around the world. Several museums offer online exhibitions, such as the Smithsonian Museum in the US, the State Hermitage Museum in Russia, and the Louvre Museum in France.

VR technology improves learner motivation and mood (Dieck et al., 2018; Puig et al., 2020), enhancing participant satisfaction and enjoyment, especially with wearable devices. It helps enhance learning experiences and personalize the learning process. Nechita and Rezeanu (2019) report that multisensory-enhanced museum spaces can enhance empathy by allowing learners to experience a variety of historical settings from a first-hand perspective. Teaching in interactive VR environments provides equal or better learning outcomes compared to traditional teaching (Newman and McLean, 2004; Stull et al., 2013; Nolin et al., 2016; Frydenberg and Andone, 2018; Felnhofer et al., 2018; Dube and Ince, 2019). However, the transformative effect of immersive VR in education has mainly been reported in the medical field (Thompson, 2009; Kim et al., 2015; Kim et al., 2017; Brusamento et al., 2019; Kyaw et al., 2019; Blumstein et al., 2020). As a new technology for museum educational activities, VR can create more engaging and immersive learning experiences (Crowley et al., 2014).

During the COVID-19 pandemic, psychotherapy conducted face-to-face increased the risk of infection, creating the need for remote therapy. The role of VR in museum education became increasingly important. As VR technology provides digitally enhanced visual complementarity to artwork details (Chang et al., 2014), administrators can pull 3D digital objects from a database and place them into a virtual exhibit room where participants can zoom into areas of interest. This method enables teaching and learning in a highly interactive environment that stimulates curiosity and creative thinking, allowing museums to develop unique, meaningful educational experiences (Smith and Blunkett, 2000). It also provides an atmosphere of freedom that can help participants with psychological disorders. Some studies have explored the application of VR to enhance visitor-exhibit interactions or allow visitors to generate virtual content in an immersive environment (e.g., Hsiao et al., 2016; Koutsabasis and Vosinakis, 2018). Bettelli et al. (2020) found that the novelty of VR may appeal to adolescents, capturing their attention and encouraging them to persist in their studies, resulting in more positive perceptions. However, little research has examined the integration of VR museum education and art therapy or the use of VR to provide telepsychotherapy through online museum-education activities.

### 4.2. Suggestions for future research

This review highlights the importance of incorporating museum education practices to enhance mental health and well-being. However, it finds a lack of research on the involvement of museum education in therapy, particularly on museum education engaged in therapeutic practice that demonstrates multifaceted possibilities of combining art therapy approaches with museum education. To better mitigate the effects of COVID-19 on the mental health of diverse groups (e.g., isolation and distress), the caring role of museums is exemplified by the construction of museum VR spaces that support social interaction, engagement, stimulation, and care, providing a broader range of distance art therapy opportunities. The portability of VR technology and therapies create opportunities for museums to explore partnerships with the education and healthcare sectors to expand their collections and programs, especially for beneficiaries embarrassed about their illnesses or anxious about the treatment process. Thus, VR museum education is a new type of education, an emerging therapy, and an increasingly important research topic.

Museum education through VR teletherapy can improve quality of life and maintain or promote health. It also hints at museums’ potential role in the mental health field: even those with hearing impairments can enjoy the virtual museum experience at home. However, access to technology remains a concern, and those who may not have easy access (e.g., people from low-income communities and with disabilities) need to be considered. Therefore, interdisciplinary collaboration with art therapists and educators is an area for further exploration to understand the needs and challenges of different groups in future planning and design of museum education and ensure inclusive strategies and practices.

## 5. Conclusion

This paper studies the practice of art therapy in museum education. We summarize its activities, main functions and practical approaches. Finally, through critical reading, we propose new directions and research trends for future development in this field. Our research results fill the gap in the practice of art therapy in museum education. Readers can obtain exciting information from the data analysis of this study, which provides valuable reference and help for future researchers.

## Author contributions

CZ contributed to the overall framework for the manuscripts as well as discussion sections. ZW contributed to the literature review and the writing of the manuscript. YG contributed to the paper revision. All authors reviewed the manuscript, contributed to the article, and approved the submitted version.

## Funding

This study was supported by the Open Research Fund of the College of Teacher Education, Zhejiang Normal University, grant no. jykf22046.

## Conflict of interest

The authors declare that the research was conducted in the absence of any commercial or financial relationships that could be construed as a potential conflict of interest.

## Publisher’s note

All claims expressed in this article are solely those of the authors and do not necessarily represent those of their affiliated organizations, or those of the publisher, the editors and the reviewers. Any product that may be evaluated in this article, or claim that may be made by its manufacturer, is not guaranteed or endorsed by the publisher.
